# Projection of new thresholds for hypertension to outpatient clinic patients and impact of risk factors: a cross-sectional study

**DOI:** 10.1590/1516-3180.2019.0111220719

**Published:** 2019-10-31

**Authors:** Umit Aydogan, Yusuf Cetin Doganer, Asli Ebiloglu, Deniz Engin Gok, Ebru Cirpan, Kenan Saglam

**Affiliations:** I MD. Associate Professor, Department of Family Medicine, Gülhane Faculty of Medicine, University of Health Sciences, Ankara, Turkey.; II MD. Associate Professor, Department of Family Medicine, Gülhane Faculty of Medicine, University of Health Sciences, Ankara, Turkey.; III MD. Family Physician, Oran Outpatient Clinic, Primary Care and Family Health Center, Ankara, Turkey.; IV MD. Endocrinologist, Department of Endocrinology and Metabolic Diseases, ARTE Surgery Hospital, Ankara, Turkey.; V MD. Family Physician, Foca Naval Base Outpatient Clinic, Primary Care and Family Health Center, Izmir, Turkey.; VI MD. Professor, Department of Internal Medicine, Gülhane Faculty of Medicine, University of Health Sciences, Ankara, Turkey

**Keywords:** Blood pressure, Hypertension, Guideline

## Abstract

**BACKGROUND:**

The 2017 American College of Cardiology (ACC)/American Heart Association (AHA) guidelines on hypertension management recommend new stage 1 hypertension thresholds (130-139/80-89 mmHg) for starting antihypertensive treatment.

**OBJECTIVE:**

To analyze the impact of the 2017 ACC/AHA guidelines on patients’ diagnoses within daily practice, in comparison with management using the 2018 European hypertension guidelines, regarding the new thresholds.

**DESIGN AND SETTING:**

Cross-sectional study conducted in a hypertension outpatient clinic at a tertiary-level public hospital.

**METHODS:**

The diagnosis of hypertension was defined separately using each guideline. The participants were patients who were attending the hypertension clinic, who were evaluated using the thresholds of two guidelines, based on cardiovascular risk factors, including age, gender, smoking status, diabetes mellitus, dyslipidemia, obesity, osteoporosis, chronic renal failure and family history of hypertension.

**RESULTS:**

After adapting the guidelines to the blood pressure values of our sample, 74.5% (n = 277) of the patients were diagnosed as hypertensive according to the blood pressure classification of the European Society of Cardiology (ESC) guidelines published in 2018, while 91.1% (n = 339) of the patients were hypertensive according to the new 2017 ACC/AHA guidelines. Multivariate regression analysis revealed that the significant demographic and cardiovascular risk factors associated with hypertension, based on the 2018 European Society of Hypertension (ESH)/ESC guidelines, were age (odds ratio, OR: 1.027; 95% confidence interval, CI: 1.001-1.054; P = 0.042), obesity (OR: 4.534; 95% CI: 1.830-11.237; P = 0.001) and family history of hypertension (OR: 2.199; 95% CI: 1.252-3.862; P = 0.006).

**CONCLUSIONS:**

The factors associated with the definition of hypertension may vary through changing the threshold values.

## INTRODUCTION

Hypertension is one of the leading public health problems in both developed and developing countries. Studies have shown that cardiovascular morbidity and mortality are closely related to systolic and diastolic blood pressure. Up-to-date guidelines are needed in order to achieve the targets that have been accepted as international standards for diagnosing and treating hypertension. Although the guidelines are not decisive in themselves alone, they assist physicians in the approach that they take towards hypertension.^1^

In 2017, the American College of Cardiology (ACC) and the American Heart Association (AHA) released new guidelines for prevention, detection, evaluation and management of hypertension in adults. The new guidelines lower the threshold for the diagnosis of hypertension and target blood pressure levels of 130/80 mmHg in the general population. The new classification will add a large number of patients who will now be diagnosed as hypertensive, whose blood pressure was previously considered to be within the normal range.^2^

The 2017 ACC/AHA guidelines differ from the criteria of the Eighth Joint National Committee (JNC-8) report, which was published in 2014.^3^ The new guidelines have developed a more aggressive approach that can be summarized as three main strands: (1) The threshold for defining hypertension has been decreased from 140/90 mmHg to 130/80 mmHg; (2) Independent of cardiovascular risk factors and blood pressure levels, the blood pressure target value has been set to < 130/80 mmHg; and (3) Selection of two antihypertensive drugs for patients with a blood pressure of 140/90 mmHg and over has been adopted (which comprises stage 2 hypertension according to the 2017 ACC/AHA guidelines, or stage 1 hypertension according to other guidelines).^4^ The new guidelines emphasize that cardiovascular disease, diabetes or a risk of more than 10% of developing cardiovascular disease within 10 years, are as important as the blood pressure values in treating hypertension. Other points of particular interest in the new guidelines are the importance placed on home blood pressure measurement and teamwork in hypertension management.^5^

Unlike the 2017 ACC/AHA guidelines, the 2013 guidelines of the European Society of Hypertension (ESH) and European Society of Cardiology (ESC) defined the threshold for stage 1 hypertension and for starting pharmacological treatment as 140-159 mmHg of systolic blood pressure (SBP) or 90-99 mmHg of diastolic blood pressure (DBP).^6^ The 2018 ESH/ESC hypertension guidelines have now also been released and the blood pressure thresholds for classifying hypertension remain the same as in the previous European guidelines.^7^

Threshold values for identifying and classifying the diagnosis of hypertension, and for determining the time to start pharmacological treatment, the target values and the treatment strategies, are important for community health and for healthcare costs. In the present study, these two current guidelines, based on different threshold values for diagnosing hypertension and on different classification tables, were evaluated through our sample. In addition, the association between demographic and cardiovascular risk factors and hypertension was examined based on the diagnostic threshold values for hypertension that are accepted in each of the two guidelines.

## OBJECTIVE

The objective of this study was to analyze the impact of each threshold, i.e. those accepted by 2017 ACC/AHA guidelines and the 2018 European hypertension guidelines, on patients’ diagnoses. In addition, we sought to ascertain the associations between the cardiovascular risk factors and each of the thresholds.

## METHODS

### Design and setting

This observational cross-sectional study was conducted at Gülhane Educational and Research Hospital, Ankara, Turkey.

### Participants, variables and data sources

A total of 437 consecutive patients who had been admitted to the hypertension outpatient clinic with a diagnosis of hypertension at the baseline assessment were recruited for this study. The baseline SBP/DBP values of this sample, from among all the enrolled patients for whom blood pressures were evaluated between 1990 and 2010, were extracted from the patients’ medical files. Patients with mental disorders and malignancies, and those younger than 18 years, were excluded from the study. After the initial evaluation of inclusion criteria and after excluding patients with deficient laboratory results, a total of 372 participants (85.1%) remained enrolled in the study.

All the study variables including the blood pressure measurement were obtained at the baseline assessment on the patients. The personal characteristics surveyed included the patients’ age group (≤ 45 years versus 46-65 years versus > 60 years), sex, obesity (body mass index, BMI ≥ 30 kg/m^2^ versus BMI < 30 kg/m^2^), smoking status (current smoker versus others) and family history of hypertension (yes versus no). Presence of any of the following diseases was also assessed: coronary artery disease (CAD), both types of diabetes mellitus (DM), dyslipidemia and chronic kidney disease (CKD). Presence of comorbidities was ascertained according to self-reports from the study participants at the baseline evaluation. The patients’ laboratory values were obtained from the hospital’s electronic biochemistry data service and from the patients’ files.

### Ethical considerations

The study protocol was approved by the Institutional Review Board of the Gülhane Education and Research Hospital of Ankara, Turkey (no. 1491-676-10/1539; date: February 19, 2010). This study was conducted in accordance with the principles of the Declaration of Helsinki. The researchers also guaranteed that the participants’ identities and related health records would be kept confidential.

### Statistical analysis

The patient groups were categorized according to the thresholds for the diagnosis of hypertension indicated by the two sets of guidelines. The statistical analysis was carried out using the Statistical Package for the Social Sciences (SPSS) software (version 22.0; SPSS Inc., Chicago, IL, USA).

Descriptive statistics were presented as percentages for categorical variables and as the mean ± standard deviation (SD) for continuous variables. All the continuous variables, including age, growth factor receptor (GFR) level, total cholesterol level, BMI and blood pressure values, were analyzed for normal distribution using the Kolmogorov-Smirnov test and were found to be normally distributed. Comparison of categorical variables was performed using the chi-square test.

Binary logistic regression modeling was used to examine the association between cardiovascular risk factors and the diagnosis of hypertension based on these two sets of guidelines, while controlling for and including all other variables.

The results were presented as odds ratios (OR) and 95% confidence intervals (95% CI). The data were considered to be statistically significant at P-values < 0.05.

## RESULTS

A total of 372 patients with a mean age of 62.36 ± 11.45 years (range: 25-88) were enrolled in this study. Just over three-fourths (76.3%; n = 284) of the study participants were female. Among all the patients, 43.3% (n = 161) were in the age group > 65 years, while 48.7% (n = 181) were in the age group 46-65 years. Only a small proportion of the patients (14%) were smoking currently. Other cardiovascular risk factors and comorbid diseases are shown in Table 1.

**Table 1.** Comparison of predictors for the diagnosis of hypertension according to the 2018 European Society of Hypertension and the European Society of Cardiology guidelines

	All % (n)	Hypertension (-) % (n)	Hypertension (+) % (n)	P*
Sex				0.895
Female	76.3 (284)	76.8 (73)	76.2 (211)	
Smoking status				0.924
Current smoker	14.0 (52)	13.7 (13)	14.1 (39)	
Diabetes mellitus				0.118
Yes	26.1 (97)	20.0 (19)	28.2 (78)	
Coronary artery disease				0.864
Yes	13.2 (49)	13.7 (13)	13.0 (36)	
Age groups				0.787
< 45 years	8.1 (30)	9.5 (9)	7.6 (21)	
46-65 years	48.7 (181)	49.5 (47)	48.4 (134)	
> 65 years	43.3 (161)	41.1 (39)	44.0 (122)	
Family history				0.013
Yes	46.8 (174)	35.8 (34)	50.5 (140)	
Obesity				0.002
Yes	22.0 (82)	10.5 (10)	26.0 (72)	
Osteoporosis				0.030
Yes	13.4 (50)	20.0 (19)	11.2 (31)	
Dyslipidemia				0.177
Yes	44.9 (167)	38.9 (37)	46.9 (130)	
Glomerular filtration rate (GFR) classes (ml/min/m^2^)				0.761
GFR ≥ 90	28.9 (93)	25.6 (20)	29.9 (73)	
GFR 60-89	58.1 (187)	60.3 (47)	57.4 (140)	
GFR 30-59	13.0 (42)	14.1 (11)	12.7 (31)	

*Chi-square test.

**Figure 1.**
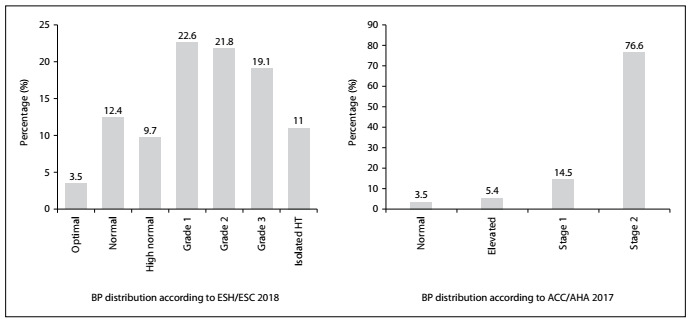
Classification of blood pressure (BP) values according to the 2017 American College of Cardiology/American Heart Association (ACC/AHA) and the 2018 European Society of Hypertension and the European Society of Cardiology (ESH/ESC) hypertension (HT) guidelines.

In the whole study group, 74.5% (n = 277) of the patients were diagnosed as presenting hypertension according to the ESC guidelines for blood pressure classification (SBP/DBP > 140/90 mmHg), published in 2018. Conversely, 91.1% (n = 339) of the patient sample were hypertensive according to the 2017 ACC/AHA guidelines for blood pressure classification (SBP/DBP > 130/80 mmHg), published in 2017. Based on the 2018 ESH/ESC guidelines for blood pressure classification, 22.6% of the patients were in stage 1, 21.8% in stage 2 and 19.1% in stage 3 group. However, based on the 2017 ACC/AHA guidelines for blood pressure classification, 14.5% were in stage 1 and 76.6% were in stage 2 (Figure 1).

Using the definition of the 2018 ESH/ESC guidelines, and comparing the patients with and without hypertension, we found that the patients in the hypertension group had more family history of hypertension (50.5%, n = 140 versus 35.8%, n = 34; P = 0.013). Among the patients with hypertension, 26.0% (n = 72) were obese, while this was observed in 10.5% (n = 10) of the patients without hypertension (P = 0.002). Regarding osteoporosis, the number of patients presenting this in the group with hypertension (11.2%, n = 31) was significantly lower than the number of patients with osteoporosis in the group without hypertension (20%, n = 19).

In accordance with the definition of the 2018 ESH/ESC guidelines, comparing the patients who had hypertension with those who did not, although the patients in the hypertension group were older and more frequently presented obesity, no statistically significant difference was detected based on cardiovascular risk factors and comorbidities (Table 1). According to the results from the univariate analysis, there were no significant variables associated with the diagnoses of hypertension that were defined in the 2017 ACC/AHA guidelines (Table 2).

**Table 2.** Comparison of predictors for the diagnosis of hypertension according to the 2017 American College of Cardiology/American Heart Association guidelines

	All % (n)	Hypertension (-) % (n)	Hypertension (+) % (n)	P
Age groups				0.056
< 45 years	8.1 (30)	18.2 (6)	7.1 (24)	
46-65 years	48.7 (181)	36.4 (12)	49.9 (169)	
> 65 years	43.3 (161)	45.5 (15)	43.1 (146)	
Sex				0.438
Female	76.3 (284)	81.8 (27)	75.8 (257)	
Smoking				0.747
Current smoker	14.0 (52)	12.1 (4)	14.2 (48)	
Diabetes mellitus				0.505
Yes	26.1 (97)	21.2 (7)	26.5 (90)	
Coronary artery disease				0.468
Yes	13.2 (49)	9.1 (3)	13.6 (46)	
Family history				0.209
Yes	46.8 (174)	36.4 (12)	47.8 (162)	
Obesity				0.060
Yes	22.0 (82)	9.1 (3)	23.3 (79)	
Osteoporosis				0.816
Yes	13.4 (50)	12.1 (4)	13.6 (46)	
Dyslipidemia				0.162
Yes	44.9 (167)	33.3 (11)	46.0 (156)	
Glomerular filtration rate (GFR) classes (ml/min/m^2^)				0.413
GFR ≥ 90	28.9(93)	22.2 (4)	29.3 (89)	
GFR 60-89	58.1 (187)	72.2 (13)	57.2 (174)	
GFR 30-59	13.0 (42)	56 (1)	13.5 (41)	

*Chi-square test analysis.

The results from the multivariate logistic regression analysis revealed that the significant demographic and cardiovascular risk factors associated with the diagnosis of hypertension according to the 2018 ESH/ESC guidelines were the following: age (OR: 1.027; 95% CI: 1.001-1.054; P = 0.042), obesity (OR: 4.534; 95% CI: 1.830-11.237; P = 0.001) and family history of hypertension (OR: 2.199; 95% CI: 1.252-3.862; P = 0.006). According to the regression analysis, no significant difference was detected in terms of the association between cardiovascular risk factors and the diagnosis of hypertension according to the 2017 ACC/AHA guidelines (Table 3).

**Table 3.** Multivariate regression analysis on associations of variables with the diagnosis of hypertension according to the 2018 European Society of Hypertension and the European Society of Cardiology guidelines and the 2017 American College of Cardiology/American Heart Association guidelines

Variables	2018 European Society of Hypertension and the European Society of Cardiology hypertension definition		
	Odds ratio	95% confidence interval	P
Sex (male)	1.167	0.621-2.194	0.631
Age	1.027	1.001-1.054	0.042
Coronary artery disease	0.829	0.399-1.722	0.615
Diabetes mellitus	1.182	0.613-2.278	0.618
Glomerular filtration rate	1.008	0.993-1.024	0.298
Smoking	1.518	0.680-3.389	0.308
Obesity	4.534	1.830-11.237	0.001
Dyslipidemia	0.892	0.513-1.552	0.686
Family history of hypertension	2.199	1.252-3.862	0.006
	2017 American College of Cardiology/American Heart Association hypertension definition		
Sex (male)	1.777	0.488-6.466	0.383
Age	1.042	0.995-1.091	0.082
Coronary artery disease	0.814	0.215-3.082	0.762
Diabetes mellitus	0.890	0.264-3.006	0.852
Glomerular filtration rate	1.004	0.975-1.034	0.795
Smoking	4.351	0.525-36.052	0.173
Obesity	1.336	0.001-2.005	0.997
Dyslipidemia	0.730	0.266-2.007	0.542
Family history of hypertension	2.793	0.927-8.420	0.068

## DISCUSSION

It has been predicted that, through lowering the threshold for making the diagnosis of hypertension to 130/80 mmHg, the 2017 ACC/AHA guidelines will increase the number of patients who will be diagnosed with hypertension and need treatment. The proportion of our sample that was not hypertensive using the 2018 ESH/ESC guideline thresholds was approximately three times higher (25.5% versus 8.9%) than it was using the 2017 ACC/AHA guideline thresholds. Compared with the 2018 ­ESH/­ESC guidelines, the number of hypertensive patients according to the 2017 ACC/AHA guidelines was 16.6% higher in our study sample. Age, obesity and family history of hypertension were significant variables according to the 2018 ESH/ESC guidelines, but use of the 2017 ACC/AHA guidelines did not give rise to any significant change among the factors associated with the diagnosis of hypertension, defined through these two different sets of guidelines.

The evidence supporting the lowering of the hypertension thresholds came from a meta-analysis on randomized controlled trial (RCTs) published in The Lancet in 2016, particularly from the data of the Systolic Blood Pressure Intervention Trial (SPRINT), in relation to antihypertensive drug treatment.^8^^,^^9^ According to this meta-analysis in The Lancet, a reduction of about 25% in blood pressure was effective in preventing the development of cardiovascular events in patients with SBP of 130 mmHg and above. However, even though SPRINT is a well-organized study, some notable concerns remain when its results are adapted for use in guidelines or real-life daily practice. The beneficial results shown by studies that have early termination may sometimes be greater than should be expected.^8^

The difference between the 2017 ACC/AHA guidelines and the previous guidelines means that the number of hypertensive patients in the United States is expected to increase from 32% to 46%, simply through changing the definition of hypertension. Moreover, the target blood pressure has decreased along with the lowering of the diagnostic threshold. According to the authors of the 2017 ACC/AHA guidelines, the prevalence of hypertension was expected to increase significantly through these guidelines. However, they claimed that early diagnosing of cardiovascular events may become possible and awareness of hypertension among patients at risk will increase over the course of these individuals’ future lives.^10^

In addition to hypertension, conditions of accompanying cardiovascular disease or diabetes, or 10-year risk of developing cardiovascular disease greater than 10%, should be taken into consideration in the management of antihypertensive treatment. The latest (2017) ACC/AHA guidelines have emphasized the importance of home blood pressure monitoring and teamwork in the management of disease.^10^ The authors of these new guidelines claimed that, since drug treatment is recommended especially in cases of clinical cardiovascular diseases such as CAD, coronary heart disease (CHD) and stroke, or in cases with a risk of developing cardiovascular disease greater than 10%, in patients with stage 1 blood pressure values (130-139/80-89 mmHg), the new classification will not increase antihypertensive drug use.^2^ However, other physicians have contested this assumption.^11^

The new blood pressure classification proposed in the 2017 ACC/AHA guidelines has been adapted to hypertension studies in different countries. Application of the threshold value of 130/80 mmHg to the latest national Chinese research data showed that the hypertension rate rose from 25% to 50%. The proportion of patients requiring medication in the Chinese population was 2.0% in the general population and 5.5% in the geriatric patient population according to the 2017 ACC/AHA guidelines.^12^

Based on the threshold of ≥ 140/90 mmHg, the current prevalence of hypertension in India is approximately 28.9% in both men and women. In the Indian population, in which there are interactions with various social, cultural and economic factors, hypertension management has become quite difficult with the lower blood pressure values that have redefined hypertension in the new 2017 ACC/AHA guidelines.^13^ It seems to be taking time in India for implementation of another emphasis of the 2017 ­ACC/­AHA guidelines, i.e. widespread adoption of out-of-office blood pressure measurement, across the country. The current situation in which the vast majority of the cost of medicines in India purchased through personal budgets is refunded also increases concern about the increased risk of antihypertensive drug usage. Moreover, the target values of the Indian hypertension guidelines published in 2013 have not yet been fully met in clinical practice.^14^ In this regard, achieving the lower target values of the 2017 ACC/AHA guidelines does not seem applicable to daily practice in India.^15^ In a study involving 6106 adults randomized from rural areas of India, a 14% increase in the number of stage 1 hypertension patients was observed, with re-evaluation of 2815 individuals according to the new thresholds.^14^^,^^16^

Another factor affecting the international generalizability of the new guidelines, which basically arose from the SPRINT study data, is blood pressure differences based on ethnicity disparities between countries. Since the proportion of East Asian ethnicity in the SPRINT trial was very low (< 2%), it is doubtful whether the results can be generalized to countries like Taiwan.^4^

In our study, the patients who enrolled at the hypertension outpatient clinic were evaluated regarding blood pressure thresholds, using both guidelines separately. From this perspective, the proportion of hypertensive patients increased from 74.5% (2018 ESH/ESC guidelines) to 91.1% (2017 ACC/AHA guidelines) in our patient sample. This increase in the proportion of hypertension diagnoses, of 16.6 percentage points, which was caused by lowering the threshold value to 130/80 mmHg, is quite spectacular. Projection of the new guidelines into various different communities has led to predictions that increases in the numbers of hypertensive patients of 14% in the United States,^10^ 25% in China^12^ and 14% in rural India^13^ will be observed. It has been estimated that the differences in the rates of increase are caused by differences in sample selection, ethnicity and cultural lifestyles.

Another noteworthy point in the present study was that significant associations were detected between the presence of hypertension and the variables of family history of hypertension, obesity and age, according to the 2018 ESH/ESC guidelines. However, no significant relationship between the presence of hypertension and these variables was observed regarding the 2017 ACC/AHA guidelines, although age and family history of hypertension were close to being significant. The results from this analysis indicate that the demographic and cardiovascular factors that are effective for making the diagnosis of hypertension might vary with the change in hypertension threshold values.

Our study conducted in Turkey can be considered to be a pilot reflecting the thresholds of the new 2017 ACC/AHA guidelines in our society. However, these increased rates of hypertension cannot be generalized to the entire Turkish population because our study had some limitations, including its small sample size and the structure of the outpatient clinic studied, which only serves hypertensive patients. Furthermore, the number of female patients evaluated was higher than the number of male patients.

## CONCLUSIONS

The decreases in hypertension threshold values proposed through the 2017 ACC/AHA guidelines have increased the number of patients diagnosed with hypertension worldwide, including in Turkey. This increase was by 16.6 percentage points in our sample.

We consider that updates to the relationship between cardiovascular risk factors and the diagnosis of hypertension through the change in threshold values may come to be presented in the near future, consequent to ongoing studies in various countries worldwide. In the current study, age, obesity and family history of hypertension were significantly associated with the diagnosis of hypertension according to the 2018 ESC guidelines, while no relationship was detected between cardiovascular risk factors and the diagnosis of hypertension using the 2017 ACC guidelines. Studies involving higher numbers of patient samples will be effective in explaining the relationship between these risk factors and the diagnosis of hypertension.
